# Multiple Arterial Dissections and Connective Tissue Abnormalities

**DOI:** 10.3390/jcm11123264

**Published:** 2022-06-07

**Authors:** Philipp Erhart, Daniel Körfer, Susanne Dihlmann, Jia-Lu Qiao, Ingrid Hausser, Peter Ringleb, Jörg Männer, Nicola Dikow, Christian P. Schaaf, Caspar Grond-Ginsbach, Dittmar Böckler

**Affiliations:** 1 Department of Vascular and Endovascular Surgery, University Hospital of Heidelberg, 69120 Heidelberg, Germany; daniel.koerfer@med.uni-heidelberg.de (D.K.); susanne.dihlmann@med.uni-heidelberg.de (S.D.); jiaiu.qiao@stud.uni-heidelberg.de (J.-L.Q.); caspar.grond-ginsbach@med.uni-heidelberg.de (C.G.-G.); dittmar.boeckler@med.uni-heidelberg.de (D.B.); 2 Institute of Pathology, University Hospital of Heidelberg, 69120 Heidelberg, Germany; ingrid.hausser-siller@med.uni-heidelberg.de; 3 Department of Neurology, University Hospital of Heidelberg, 69120 Heidelberg, Germany; peter.arthur.ringleb@med.uni-heidelberg.de; 4 Institute of Anatomy and Embryology, UMG, University of Göttingen, 37075 Göttingen, Germany; jmaenne@gwdg.de; 5 Institute of Human Genetics, Heidelberg University, 69120 Heidelberg, Germany; nicola.dikow@med.uni-heidelberg.de (N.D.); christian.schaaf@med.uni-heidelberg.de (C.P.S.)

**Keywords:** dissection, carotid artery, genetics, connective tissue disease, Marfan syndrome, Ehlers–Danlos syndrome

## Abstract

Background: Although patients with multiple arterial dissections in distinct arterial regions rarely present with known connective tissue syndromes, we hypothesized that mild connective tissue abnormalities are common findings in these patients. Methods: From a consecutive register of 322 patients with cervical artery dissection (CeAD), we identified and analyzed 4 patients with a history of additional dissections in other vascular beds. In three patients, dermal connective tissue was examined by electron microscopy. DNA from all four patients was studied by whole-exome sequencing and copy number variation (CNV) analysis. Results: The collagen fibers of dermal biopsies were pathologic in all three analyzed patients. One patient carried a CNV disrupting the *COL3A1* and *COL5A2* genes (vascular or hypermobility type of Ehlers–Danlos syndrome), and another patient a CNV in *MYH11* (familial thoracic aortic aneurysms and dissections). The third patient carried a missense substitution in *COL5A2*. Conclusion: Three patients showed morphologic alterations of the dermal connective tissue, and two patients carried pathogenic variants in genes associated with arterial connective tissue dysfunction. The findings suggest that genetic testing should be recommended after recurrent arterial dissections, independently of apparent phenotypical signs of connective tissue disorders.

## 1. Introduction

Arterial dissections occur when blood enters between the layers of the arterial wall. Depending on arterial location, acute dissection is an emergency event with high morbidity and mortality [1,2,3]. The pathogenesis of spontaneous dissection is largely unclear and may be different across the vascular system. Several risk factors are associated with arterial dissection, including smoking, arterial hypertension, fibromuscular dysplasia, arterial tortuosity and hereditary connective tissue disorders [4]. Genetic research and testing identified candidate genes for the hereditary thoracic aortic aneurysm and dissection disease complex (TAAD). In clinical routine, genetic panel testing considers a selection of genes with highly penetrant mutations for TAAD [5]. Mendelian disorders seem to contribute less to dissections in other vascular locations (cervical, visceral or coronary arteries), but genetic variants in TAAD may be involved. To date, no genetic etiologies have been established for patients with multiple arterial dissections in different vascular locations. In the current study, we performed genomic analyses in four selected patients with independently occurring dissections in both the aorta and cervical arteries.

## 2. Materials and Methods

Patients with a history of previous or subsequent aortic dissection were identified in a consecutive register of 282 patients with cervical artery dissection (CeAD) recruited between 1995 and 2018 at the Department of Neurology of the University Hospital Heidelberg and in 40 additional CeAD patients that were referred to the department from other hospitals. Anamnesis and clinical examination were performed in all patients from the Heidelberg register to identify phenotypical signs of connective tissue dysfunction including joint hypermobility and skin anomalies. Clinical data from the other 40 patients were extracted from medical reports and—if required—completed by contacts with the referring medical center.

Skin biopsies were taken by deep knife skin biopsy from the outer aspect of the upper arm close to the elbow and analyzed by electron microscopy after fixation in glutaric aldehyde/OsO_4_, resin embedding, ultrathin sectioning and staining with uranyl acetate/lead citrate.

Peripheral blood was used for DNA extraction after patients gave their written consent to the study protocol. Genetic studies included analysis of Copy Number Variation (CNV) and whole-exome sequencing analysis. CNV analysis was performed on Affymetrix 6.0 high-density microarrays in two patients by PennCNV analysis and subsequent validation by visual inspection of the CNV findings and molecular validation as described before [6,7]. Exome sequencing was performed on a Genome Analyzer IIx system (Illumina Inc., San Diego, CA, USA) after in-solution enrichment of exonic sequences (SureSelect Human All Exon 38 Mb kit, Agilent Technologies Germany GmbH & Co. KG, 76337 Waldbronn, Germany). Read alignment was performed by the Burrows–Wheeler Aligner (BWA, version 0.5.8) to the human genome assembly hg19. Single-nucleotide variants and small insertions and deletions (indels) were detected with SAMtools (v 0.1.7). CNV were identified by using the program fishing CNV [8].

All genes harboring prioritized exome variants were analyzed for enrichment of predefined gene sets with the Set Distiller software (http://www.genecards.org, accessed on 1 May 2022). For the interpretation of sequence variants and CNVs, we followed the guidelines of the American College of Medical Genetics and Genomics [9,10]. Genes associated with Mendelian disorders were identified by analysis with the DAVID (Database for Annotation, Visualization and Integrated Discovery) software package (version 6.7, the MalaCards Human diseases database (http://www.malacards.org, accessed on 19 February 2022) and by PubMed search. Single-nucleotide variants causing a predicted amino acid substitution (missense variants) were analyzed with polyphen-2 (http://genetics.bwh.harvard.edu/pph2/, accessed on 1 May 2022) to predict the possible functional impact of the substitution. All genes disrupted by CNV were searched in the ohnolog database (http://ohnologs.curie.fr/cgi-bin/SearchPage.cgi, accessed on 19 February 2022) to identify strict ohnologs. CNVs covering a strict ohnolog were considered pathogenic, since ohnologs in the human genome are dosage-balanced and frequently associated with disease [11,12].

The study protocol was approved by the local ethics committee of the university hospital Heidelberg (reference number S-190/2004, amendment S-551/2018). Genetic counseling and validation were offered to the affected individuals according to the regulations of the German Genetic Diagnostics Act.

## 3. Results

Among 282 patients with carotid and/or vertebral artery dissections, we identified 3 (1.0%) patients with an independent aortic dissection event. A fourth patient with aortic dissection was identified among 40 CeAD patients who were referred to Heidelberg from external hospitals.

None of the four patients with both cervical and aortic dissections had relevant comorbidities, drug abuse or anamnestic traumas that could account for arterial dissection. All patients were male, and their age at the first onset of dissection was between 36 and 47 years. The dissections occurred as independent events in different arteries. Locations of arterial dissection and patient characteristics are provided in Table 1.

**Figure 1:**
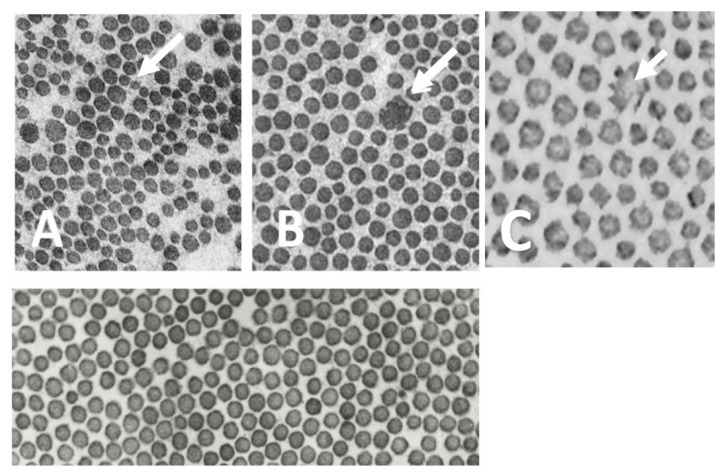
Electron microscopy of collagen fibers from skin biopsies from patient 2 (**A**), patient 3 (**B**) and patient 4 (**C**) and a healthy control individual (lower panel). Arrows indicates composite collagen fibrils. Collagen fibrils are aberrative and different in diameter. Magnification: 40,000×.

One patient showed mild clinical signs of hypermobile joints and hyper elastic skin. Three patients (patients 2, 3 and 4) gave consent for an invasive skin biopsy. All three examined skin biopsies showed structural abnormalities of skin connective tissue integrity, namely, small-caliber collagen fibrils in one patient and abnormal/composite collagen fibrils in the other two patients (Figure 1). Patient 1 had several relatives with vascular events: his father, his grandfather as well as three paternal relatives suffered from aortic events. A sister of patient 2 had an aortic dissection. No family history was documented for patients 3 and 4.

Genetic studies included next-generation sequencing to detect exome variants (performed in patients 1 and 4), as well as SNP microarray analysis to detect structural variants (Copy Number Variation, CNV, performed in patients 2 and 3). Three patients carried large CNVs covering protein-coding genes (Table 2). Patient 2 carried a large CNV covering COL*3A1* and *COL5A2*, associated with different subtypes of the Ehlers–Danlos syndrome. Patient 1 had a large duplication covering nine genes including *MYH11*, a gene associated with familial thoracic aortic aneurysms and dissections. Patient 3 carried a partial deletion of the *SERPINB2* gene, encoding Plasminogen Activator Inhibitor 2 (SV(loss):chr18:59.640.388–59.694.035; del(18)q21.33)(61489408–61543055) × 1). No large CNV that covered protein-coding genes was found in patient 4.

Whole-exome sequencing analysis was performed in patient 1 and in patient 4. Variants (frequency < 0.01%, non-Finish-Caucasian superpopulation) causing a frameshift, a stop of translation or a missense substitution with predicted damaging effect (polyphen-2 score > 0.95) are listed in the Supplementary Table S1. A single-nucleotide variant (SNV) causing an amino acid substitution (Gln856Glu) in the COL5A2 pro-peptide was suspected to be disease-related in patient 4. This missense variant was not reported before and was absent from the Genome Aggregation Database (gnomAD) and classified as a variant of uncertain significance (VUS).

## 4. Discussion

This is the first comprehensive genetic analysis of a small sample of patients with independent aortic and cervical artery dissection events. The key findings were (1) that pathogenic genetic variants for connective tissue disruption are enriched and (2) mild impairment of connective tissue components are frequently inapparent in these patients’ clinical phenotype.

All patients were young (<47 years) at disease onset. Observed genetic variants in 3 patients (CNV affecting MYH11 and COL3A1 in patients 1 and 2 as well as a missense single-nucleotide variant in COL5A2 in patient 4) are suggestive to be the underlying pathogenic mutation causing multiple dissections in young patients [13,14,15,16,17,18]. Electron microscopic examination of the skin revealed subclinical alterations of connective tissue components in patients 2 and 3.

All observed reported findings in Table 2 are rare in the human population. The duplication in patient 1 (dup(16)(p13.11)) was observed in 25 individuals of the Welcome Trust Case Control Consortium (*n* = 10,259) [19] and is listed in the NCBI database of human genomic Structural Variation (https://www.ncbi.nlm.nih.gov/dbvar, accessed on 19 February 2022). This duplication was associated with an increased risk of aortic dissection [20] and cervical artery dissection [21] by previous studies. The CNV in patient 2 is absent from dbVar and is therefore considered extremely rare. Since loss-of-function variants of *COL3A1* cause the vascular type of the Ehlers–Danlos syndrome, we consider this deletion causative of the vascular phenotype of patient 2. The *COL5A2* single-nucleotide variation found in patient 4 had a polyphen-2 score of 0.963, supporting pathogenicity. It is absent from the Genome Aggregation Database (gnomAD), which suggests that this finding is very rare.

Genetic mutations and variations are more precisely investigated for aortic pathologies, and modern panel testing of candidate genes exists to confirm known connective tissue disorders affecting the thoracic and abdominal aorta [22,23]. In these studies, genetic testing is applied to both aneurysms and dissections of the thoracic aorta (TAAD). It is assumed that almost 25% of patients with TAAD have mutations in candidate genes coding for components of the extracellular matrix (e.g., *COL3A1, FBN1, LOX*), smooth muscle cell function (e.g., *ACTA2, MYH11, MYLK, PRKG1*) or the transforming growth factor-β signaling pathway (e.g., *SMAD3, TGFB2, TGFBR1, TGFBR2*) [24]. Milewicz et al. concluded recently that in patients younger than 55 years with acute aortic dissections without known familial predisposition, disease-causing changes in one of the 11 genes for TAAD are suspected, and therefore genetic testing in these patients is recommended [25]. It should be mentioned that other genetic mutations and variants apart from the investigated candidate genes are not investigated by routine genetic panel testing, and the rate of positive findings might be even higher.

In a large heterogenous cohort, Witsch et al. reported that CeAD was associated with a four-fold increased risk of subsequent aortic dissection. Future studies need to investigate which CeAD patients are at increased risk for aortic dissection in order to provide preventive screening, genetic counselling and aggressive cardiovascular risk factor control [26].

In our study, we investigated young patients suffering from multiple arterial dissections without relevant risk factors, aneurysmal disease, familial predisposition or syndromic phenotypes. Specific genetic testing should also be performed in patients with multiple aneurysms to see genetic differences distinct from those associated with multiple arterial dissections. It is unclear if these genetic findings in our patients should be interpreted as classical hereditary connective tissue diseases, as conclusive phenotypical signs were missing.

After positive genetic testing, an interdisciplinary follow-up and familial counseling should be offered to affected patients and families.

In general, the majority of CeAD patients from the register study presented with a single CeAD event (79.0%), 4.6% with multiple CeAD, and 9.2% with subsequent CeAD events within 4 weeks [15]. An association between recurrent CeAD and connective tissue disorders could not be found [5]. As hereditary connective tissue diseases are rarely found in patients with CeAD [25], we hypothesize from our results that relevant genetic findings in young patients with multiple arterial dissections must be suspected. It is a limitation of this study that CeAD patients without metachronous arterial dissection were not genotyped as a control group. In addition, the number of investigated patients was low. A multicenter approach should examine an higher number of individuals with multiple arterial dissections for a more detailed genetical investigation.

**Figure 2:**
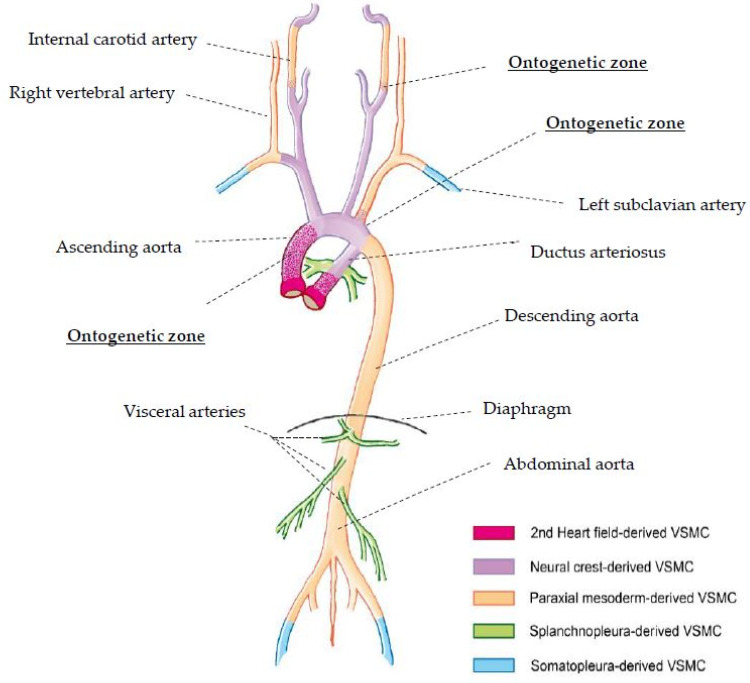
Embryologic origin of vascular smooth muscle cells (VSMCs). Ontogenetic zones are illustrated in dotted coloration and considered as predilected sites for vascular pathologies. This schematic drawing depicts the regional differences in the embryonic origin of smooth muscle cells (synthetic, contractile) forming the tunica media of the wall of the aorta and its main branches. Data are based on fate-mapping studies in mouse and chick embryos [29,30,31,32,33,34,35]. There is compelling evidence that regional differences in the embryonic origin of vascular smooth muscle cells (VSMCs) contribute to regional differences in structure, physiology and pathology of the arterial vasculature [36,37]. Boundaries between arterial tunica media segments derived from different embryonic sources seem to be predilection sites for vascular pathologies (e.g., aortic coarctation). With regard to arterial dissections, we should point to the peculiar border between the populations of mesoderm-derived (second heart field) VSMCs and neural crest-derived VSMCs within the wall of the ascending aorta (Stanford type A aortic dissection). The latter VSMC population forms the inner layers of the tunica media, whereas the former population forms the outer layers (see inset). Further boundaries between neural crest-derived segments of the arterial tunica media and mesoderm-derived (paraxial mesoderm) segments are found at the border between the aortic arch and the descending aorta (Stanford type B aortic dissection) and along the course of the internal carotid arteries (CeAD).

From embryologic investigations, it is estimated that vascular smooth muscle cells (VSMCs) and fibroblasts have distinct origins (Figure 2). In current mice embryological studies, it could be shown that vascular cells within the outer layer of the tunica media of the ascending aorta derive from the 2nd heart field (pharyngeal mesoderm), whereas the inner cell layers form from the neural crest ectomesenchyme. These transition zones (Figure 2) are referred to as ontogenetic borders and suspected to be predilection sites for vascular pathologies, such as Stanford type A or B aortic dissection and CeAD [27,28]. Multiple dissections might occur predominantly in arterial locations based on embryologic aspects or similarities in vessel wall composition.

Electron microscopy and structural analysis of dermal connective tissue integrity can support undefined genetic findings and novel mutations for vascular disease. In our case, the deletion within the gene locus of *SERPINB2* must be considered as a variant of unknown significance. From the literature, genetic variation in *SERPINB2* was associated with, e.g., gingivitis, neoplasia or rhinosinusitis [38,39]. However, it remains unclear whether the dermal connective tissue phenotype of this patient is causally related to the observed deletion in *SERBINB2*.

It is known from other studies that identical genetic alterations in connective tissue components might result in variable vascular pathologies within family members. Mazzella et al. published observations in family members with a *TGFBR2* frameshift mutation (Loeys–Dietz syndrome type 4) who suffered from diffuse vascular lesions, e.g., intracranial aneurysms, medium-caliber artery dissection, aortic and iliac aneurysms [40]. Shalata et al. detected a novel myosin light-chain kinase (*MYLK*) gene mutation in a large family suffering aortic aneurysm and dissection [41]. Due to the phenotypic variability and affection of variable vascular parts, we conclude that genetic testing in patients with multiple vascular pathologies is advisable. According to the European Society for Vascular and Endovascular Surgery clinical practice guidelines, genetic testing should be performed in TAAD individuals younger than 40 years [42]. Additionally, we suggest a careful physical examination concerning connective tissue abnormalities and genetical testing in individuals with multiple arterial dissections.

## 5. Conclusions

Our findings suggest that multiple arterial dissections per se point to hereditary connective tissue dysfunction, even though phenotypical signs might be clinically inapparent. Therefore, in these patients, genetic consultation and testing should be performed in appropriate centers. Genetic testing enables precision medicine and identifies novel mutations for arterial dissection. Based on the genetic findings, patient-specific follow-up investigations and counselling of patients and familial members can be initiated.

## Figures and Tables

**Table 1:** Patient characteristics and clinical findings.

Patient	Sex, Age *	Arterial Locations **	Phenotype	Familial History
1	m, 36	right ICA, aorta type B	normal	positive
2	m, 39	bilateral ICA, left VA, aorta type B	normal	positive
3	m, 45	left ICA, aorta type A	normal	n.d.
4	m, 47	right ICA, aorta type B	hypermobile joints	n.d.

* Age at onset of the first dissection event. ** Aortic dissections were classified according to the Stanford classification; fam hist: family history of arterial events (dissections, ruptured aneurysm); ICA: internal carotid artery; VA: vertebral artery dissection; n.d.: not documented.

**Table 2:** Genetic findings. Copy number variants were detected in three patients. Underlined genes are ohnolog genes.

Patient	Structural Variant (SV)/Exome Variant (EV)	Nomenclature ISCN2020/HGVS	ACMG Classification	Affected Genes
1	SV (gain): chr16: 14,916,662–16,306,102	dup(16)(p13.11) NC_000012.11:g.14916662_16306102dup	Likely pathogenic	*MPV17L*, *C16orf45*, *MARF1*, *NDE1*, *MYH11*, *FOPNL*, *ABCC1*, *ABCC6*, *NOMO3*
2	SV (loss): chr2:189.109.859–189.763.802	del(2)(q32.2)(189401614-190055557) × 1	Pathogenic	*GULP1*, *DIRC1*, *COL3A1*, *COL5A2*
4	EV: chr2:189.917.732 G/C	gDNA: Chr2(GRCh37):g.189917732G > C	Variant of uncertain significance	*COL5A2*, amino acid missense substitution Gln856Glu

The genetic findings were classified and interpreted according to the American College of Medical Genetics and Genomics (ACMG) [9,10], and nomenclature was based on the International System for Human Cytogenomic Nomenclature (ISCN). Large structural variants were observed in patients 1 and 2. These large CNVs affected multiple protein-coding genes. Ohnolog genes involved in connective tissue structure or synthesis are underlined. In patient 4, a single-nucleotide variant was detected. All findings were located on genome assembly GRCh37 (hg19) from the Genome Reference Consortium. CNV = Copy Number Variation; SNV = Single-Nucleotide Variant.

## Data Availability

The data are available in the manuscript and on personal request to the corresponding author.

## Supplementary Materials

The following supporting information can be downloaded at: https://www.mdpi.com/article/10.3390/jcm11123264/s1. Table S1: Whole exome sequencing findings in patient 1 and patient 4.
