# Supplementary material for: Multiple Arterial Dissections and Connective Tissue Abnormalities
Source: J Clin Med. 2022 Jun 7;11(12):3264. doi: 10.3390/jcm11123264 (PMC9224905; doi:10.3390/jcm11123264)
Supplement: Supplementary file 1 [file jcm-11-03264-s001.zip › jcm-1705547-supplementary.pdf]

Supplementary Table S1. Whole exome sequencing findings in patient 1 and patient 4.

Patient	Gene	Transcript	OMIM disease (mode of inheritance)	polyphen-2	SIFT
1	ANKRD24	uc010dtt.1,ANKRD24,+,1483G->A,0,non-syn,Glu495Lys		0.996	0.17
1	ASB16	uc002ifl.1,ASB16,+,1237C->T,0,non-syn,His413Tyr		0.999	0
1	ASPM	uc001gtw.4,ASPM,-,2358T->A,2,non-syn,Lys786Asn	Microcephaly 5, primary (ar)	0.977	0.15
1	BRAT1	uc003smi.3,BRAT1,-,107A->T,1,non-syn,Phe36Tyr	Neurodevelopmental disorder with cerebellar atrophy (ar)	0.991	0
1	BRD8	uc011cyl.2,BRD8,-,459_459delT,2,frame-shift,137501672,-T,del			
1	C9orf89	uc004atd.3,C9orf89,+,224G->A,1,non-syn,Arg75Gln		0.954	0.02
1	CADM3	uc001ftk.2,CADM3,+,1243A->T,0,non-syn,Ile415Phe	Charcot-Marie-Tooth disease, axonal, type 2FF (ad)	0.999	0.01
1	CALCR	uc022ahi.1,CALCR,-,1043A->G,1,non-syn,Leu348Pro		0.996	0
1	CBS	uc002zcs.1,CBS,-,853A->G,0,non-syn,Trp285Arg	Homocystinuria (ar)	0.996	0
1	CEP250	uc021wco.1,CEP250,+,2156G->A,1,non-syn,Arg719Gln	Cone-rod dystrophy and hearing loss 2 (ar)	0.997	0.13
1	CHRM3	uc021plc.1,CHRM3,+,262C->G,0,non-syn,Leu88Val	Prune belly syndrome (ar)	0.996	0
1	CRIPAK	uc003gdf.2,CRIPAK,+,size_31,2,frame-shift,1388775,indellength=31,ins			
1	CXorf23	uc004czo.3,CXorf23,-,692T->C,1,non-syn,Tyr231Cys		0.971	
1	DST	uc003pcy.4,DST,-,11508C->A,2,non-syn,Trp3836Cys	Epidermolysis bullosa simplex 3 (ar)	0.999	0.01
1	FBXW5	uc004cjz.3,FBXW5,-,758T->C,1,non-syn,Gln253Arg		0.979	0.25
1	GPER	uc010ksd.1,GPER,+,802T->G,0,non-syn,Phe268Val		0.999	0
1	KCNMB2	uc031scj.1,KCNMB2,+,316C->A,0,non-syn,Arg106Ser		0.977	0.15
1	LRWD1	uc003uzn.3,LRWD1,+,433G->C,0,non-syn,Val145Leu		0.987	
1	LTBP4	uc002ook.1,LTBP4,+,2062G->A,0,non-syn,Gly688Ser	Cutis laxa, type IC (ar)	0.999	0.64
1	LUM	uc001tbm.3,LUM,-,929C->T,1,non-syn,Arg310His		0.997	0
1	NDUFAF7	uc002rqa.4,NDUFAF7,+,655G->A,0,non-syn,Asp219Asn		1	0
1	NDUFS2	uc001fyv.3,NDUFS2,+,805C->T,0,non-syn,Arg269Stop	Mitochondrial complex I deficiency, nuclear type 6 (ar)		0.15
1	NOC3L	uc001kjq.1,NOC3L,-,1243C->A,0,non-syn,Glu415Stop			0.78
1	NPHS1	uc002oby.3,NPHS1,-,3619_3619delC,0,frame-shift,36317522,-C,del	Nephrotic syndrome, type 1 (ar)		
1	PLEKHH2	uc002rte.3,PLEKHH2,+,1663T->C,0,non-syn,Tyr555His		0.999	0
1	PPL	uc002cyd.1,PPL,-,1456G->A,0,non-syn,Gln486Stop			0.98
1	PTCHD2	uc001ash.4,PTCHD2,+,1781T->C,1,non-syn,Met594Thr		0.999	0.37

1	RALGAPA1	uc001wti.3,RALGAPA1,-,5919T->A,2,non-syn,Arg1973Ser	Neurodevelopmental disorder with hypotonia (ar)	0.994	0.06
1	RNF126	uc010drs.3,RNF126,-,416C->T,1,non-syn,Arg139Gln		0.997	0.63
1	SART1	uc001ogl.3,SART1,+,1558G->A,0,non-syn,Asp520Asn		0.994	0
1	SEC23B	uc010zsb.2,SEC23B,+,1566_1566delG,2,frame-shift,18523770,-G,del	Dyserythropoietic anemia, congenital, type II (ar)		
1	SLCO1A2	uc001rer.3,SLCO1A2,-,269C->T,1,non-syn,Gly90Asp		0.995	0
1	SRPX	uc004ddy.2,SRPX,-,1272_1273delCC,0,frame-shift,38009085,-CC,del			
1	SRRM2	uc002crj.1,SRRM2,+,622G->A,0,non-syn,Gly208Arg		0.998	0.04
1	SSX1	uc004djb.1,SSX1,+,64A->C,0,non-syn,Ser22Arg		0.962	0.84
1	STAM	uc001ipj.2,STAM,+,727A->G,0,non-syn,Ser243Gly		0.981	0.09
1	STON2	uc010tvu.3,STON2,-,1594T->C,0,non-syn,Thr532Ala		0.997	0.01
1	SV2B	uc002bqt.3,SV2B,+,521G->A,1,non-syn,Arg174Lys		0.999	0
1	TEK	uc003zqi.4,TEK,+,2025G->C,2,non-syn,Lys675Asn	Venous malformations (ad)	0.998	0.24
1	TIE1	uc010okc.2,TIE1,+,815G->A,1,non-syn,Cys272Tyr	Lymphatic malformation 11 (ad)	0.996	0
1	TSPAN10	uc021ufc.1,TSPAN10,+,339_340insGTCA,0,frame-shift,79612206,+GTCA,ins			
1	WNK2	uc004ati.1,WNK2,+,5336C->T,1,non-syn,Ala1779Val		0.993	0.38
1	ZNF721	uc003gaf.5,ZNF721,-,1108A->G,0,non-syn,Cys370Arg		0.992	0
4	ABP1	uc003why.1,ABP1,+,590G->A,1,non-syn,Arg197His	n.a.	1	0.01
4	C14orf166B	uc001xsx.2,C14orf166B,+,1163C->T,1,non-syn,Pro388Leu	n.a.	0.973	0.04
4	COL5A2	uc002uqk.3,COL5A2,-,2566G->C,0,non-syn,Gln856Glu	Ehlers-Danlos syndrome, classic type, 2 (ad)	0.983	
4	CUX2	uc001tsa.2,CUX2,+,2680C->T,0,non-syn,Arg894Cys	Developmental and epileptic encephalopathy (ad)	0.998	0.1
4	FAM126B	uc002uws.4,FAM126B,-,191T->C,1,non-syn,Tyr64Cys	n.a.	0.992	0
4	GPR22	uc022ajv.1,GPR22,+,410C->G,1,non-syn,Thr137Ser	Congenital disorder of glycosylation, type IIi (ar)	0.983	0.95
4	GPX2	uc021ruq.2,GPX2,-,146T->A,1,non-syn,Gln49Leu	n.a.	0.964	0.01
4	LOC440040	uc010rhy.2,LOC440040,+,784C->T,0,non-syn,Arg262Cys	n.a.	0.999	
4	LRRC16B	uc001wlj.2,LRRC16B,+,760G->A,0,non-syn,Gly254Arg	n.a.	0.998	0.6
4	OR2M7	uc010pzk.2,OR2M7,-,856T->G,0,non-syn,Asn286His	n.a.	0.999	0
4	PSG8	uc010ein.3,PSG8,-,419C->G,1,non-syn,Cys140Ser	n.a.	0.999	0
4	RNF32	uc003wms.3,RNF32,+,625C->A,0,non-syn,Leu209Met	n.a.	0.999	0

4	RP1	uc011ldy.1,RP1,+,2791G->T,0,non-syn,Glu931Stop	Retinitis pigmentosa 1 (ad, ar)		0.26
4	SLC24A5	uc001zwd.3,SLC24A5,+,18_18delC,2,frame-shift,48413258,-C,del	Albinism, oculocutaneous, type VI (ar)		
4	SLC24A7	uc003cdv.4,SLC4A7,-,1090G->A,0,non-syn,Gln364Stop			0.35
4	SMARCC1	uc003crq.2,SMARCC1,-,889G->A,0,non-syn,Arg297Cys	n.a.	0.999	0
4	TRHR	c003ymz.4,TRHR,+,1128C->A,2,non-syn,Tyr376Stop	Hypothyroidism, congenital, nongoitrous, 7 (ar)		1
4	TTN	uc031rqc.1,TTN,-,76439C->T,1,non-syn,Arg25480His	Cardiomyopathy (ad ar)	0.999	0
4	USH2A	uc001hku.1,USH2A,-,6396_6397insG,2,frame-shift,216173833,+G,ins	Usher syndrome, type 2A (ar)		
4	WDR52	uc003ead.2,WDR52,-,3064G->A,0,non-syn,Arg1022Stop	Spermatogenic failure 20 (ar)		1
4	ZNF543	uc002qoi.2,ZNF543,+,1268A->G,1,non-syn,Tyr423Cys	n.a.	0.97	0

OMIM: Online Mendelian Inheritance in Man; polyphen-2: SIFT: The Sorting Intolerant from Tolerant tool to predict the effect of coding variants on protein function. n.a. = not available
